# Impact of Crystallinity and Ni–Fe Composition on the Oxygen Evolution Performance of Ni*_x_* Fe_1‐*x*_O Electrocatalysts in Anion Exchange Membrane Water Electrolysis

**DOI:** 10.1002/cssc.202502755

**Published:** 2026-05-15

**Authors:** Atta Muhammad, Tatiana Rodriguez‐Flores, Mohsin Muhyuddin, Fabio Di Fonzo, Enrico Berretti, Alessandro Lavacchi, Carmelo Lo Vecchio, Irene Gatto, Vincenzo Baglio, Roberto Nisticò, Carlo Santoro

**Affiliations:** ^1^ Department of Materials Science University of Milano‐Bicocca Milano Italy; ^2^ Electrocatalysis and Bioelectrocatalysis Laboratory University of Milano‐Bicocca Milano Italy; ^3^ X‐Nano s.r.l. Milan Italy; ^4^ Consiglio Nazionale Delle Ricerche (CNR) Istituto di Chimica Dei Composti OrganoMetallici (ICCOM) Firenze Italy; ^5^ CNR‐ITAE Istituto di Tecnologie Avanzate per l’Energia “Nicola Giordano” Messina Italy

**Keywords:** AEM‐WE, alkaline media, NiFe oxide, oxygen evolution reaction (OER), platinum group metals (PGM)‐free electrocatalysts, rotating disk electrode (RDE)

## Abstract

Expensive platinum group metals (PGMs) are used to enhance the anodic oxygen evolution reaction (OER) kinetics, and they represent a real bottleneck in the commercialization of anion exchange membrane water electrolyzers (AEMWEs). Therefore, we present a scalable and economical homogeneous precipitation method to synthesize Ni_*x*_Fe_1‐*x*_O nanoparticles with different Ni/Fe ratios, while reducing the dependence on expensive PGM‐based electrocatalysts. The effects of Ni/Fe ratios in the synthesized Ni_*x*_Fe_1‐*x*_O, along with morphological and surface chemical characteristics, on electrocatalytic performance were thoroughly investigated with half‐cell measurements. Furthermore, critical electrode design factors, that is, ink composition and electrocatalyst loading, were scientifically investigated and optimized. Among the explored compositions, amorphous Ni_0.28_Fe_0.72_O and crystalline Ni_0.66_Fe_0.34_O exhibited superior OER activity, achieving mean overpotentials of 359 mV and 359.1 mV at 10 mA cm^−2^, respectively. This superior activity was attributed to a higher concentration of Ni^3+^ (NiOOH), a highly active compound for OER. These high‐performing samples were integrated as anodes in a lab‐scale AEMWE for device‐level evaluation. Ni_0.28_Fe_0.72_O achieved the highest performance at 80 °C, by delivering the current density of 7.81 A cm^−2^ against a cell voltage of 2.2 V. Whereas, Ni_0.66_Fe_0.34_O achieved a current density of 6.49 A cm^−2^ at 2.2 V. Both samples exhibited excellent stability during short‐term durability tests (ca. 90 h) at 1 A cm^−2^ and 80°C.

## 1 Introduction

Green hydrogen, mainly produced through water electrolyzers, is gaining recognition as a reliable and innovative energy source due to its high energy density and heating value of 141.9 KJ g^−1^, which is approximately three times higher than that of conventional fuels without any contribution to global carbon footprints [1, 2, 3].

Green hydrogen can be important in industries having difficulty in transitioning to direct decarbonization with the help of renewable electricity. Industrial processes (i.e., fertilizer and steel production) and heavy‐duty transport are two of these difficult‐to‐decarbonize sectors. Hydrogen can replace methane to produce ammonia for fertilizer production, whereas electrolytic hydrogen can replace carbon monoxide for the reduction of iron oxides [4]. Regarding heavy‐duty transportation, battery deployment is critical due to weight constraints and long traveling distances, whereas hydrogen fuel cells are more efficient [5]. Therefore, to achieve net‐zero carbon emissions, drastic measures should be taken to achieve the global demand for hydrogen, which is expected to rise from 90 million tons per year (Mtpa) in 2020 to over 500 Mtpa by 2050 [6].

Since the early 20^th^ century, hydrogen has been produced by alkaline water electrolyzers (AWEs) using nickel as electrodes and potassium hydroxide electrolyte (ca. 30 wt%) at a temperature between 80°C and 90°C [6]. Proton exchange membrane water electrolyzers (PEMWEs) present the advantages of having a compact design, dynamic operating conditions, high operating efficiencies, and quick startup and response times along with the production of pressurized (ca. 30 bar) hydrogen [7, 8, 9, 10]. However, due to acidic operational conditions, the use of critical and expensive platinum group metals (PGMs) is unavoidable [11]. More critically, worldwide deployment of PEMWEs is anticipated to be limited due to the production capacity of iridium (Ir) [12]. Anion exchange membrane water electrolyzers (AEMWEs) combine the advantages of both AWEs and PEMWEs, with the possibility of operating without the use of critical raw materials (CRMs) and pressurizing the hydrogen inside the system due to the presence of a polymeric membrane separator. AEMWEs facilitate water electrolysis through two key half‐cell reactions: oxygen evolution reaction (OER) at the anode and hydrogen evolution reaction (HER) at the cathode. As compared to HER, the four‐electron transfer OER is a thermodynamically complex and kinetically sluggish reaction; thus, it corresponds to the major burden and is considered a serious bottleneck to the overall water splitting reaction [13]. Thermodynamically, a potential of 1.23 V at 25°C is required for overall water splitting; however, practical operations owing to the high ohmic resistance and slow catalytic activity of the system require an additional ≥ 0.2 V. [14, 15, 16]. Therefore, to improve the sluggish kinetics of OER, the need for the development of a robust electrocatalyst is crucial, unlike PEMWEs, which use economically unfeasible noble metal oxides such as RuO_2_ and IrO_2_ as anode electrocatalysts and Pt‐based electrocatalysts for HER at the cathode.

AEMWEs have the advantage of using low‐cost transition metal‐based electrocatalysts for both OER and HER, not only making it economically feasible, but the use of less critically produced metals can also contribute to the scalability of hydrogen production [17, 18, 19]. Nonetheless, OER remains the limiting reaction in basic media, hence contributing higher overpotentials at 10 mA cm^−2^ and low operational durability. Therefore, it is very important to develop novel ways for fabricating OER electrocatalysts that are durable, economical, and scalable [20, 21]. Recently, NiFe‐based oxides have acquired significant attention due to their synergistic effects, adjustable stoichiometries, low cost, high availability, and superior OER performance compared to individual metal components [22, 23]. The interaction between Ni and Fe within oxides, owing to their octahedral structure, enables enhanced electron transfer and electrocatalytic activity. NiFe‐oxide electrocatalysts are known to exhibit low overpotentials for extended periods at low current density, but prolonged operational stability at high current density is challenging due to Fe segregation, structural degradation, and peeling off from the substrate [23]. Researchers have addressed this issue with quite a few techniques, such as dynamically healing the Fe active sites [24], oxyanion engineering [25], improving wettability, efficient evolution of the gas bubbles, and structural design modification of the electrocatalyst [26]. However, the implication of such complex processes is time‐consuming and limited to small‐scale modifications, which creates opportunities for improving the structural features to maximize their electrocatalytic activity and durability [27, 28]. Recently, Shi et al. conducted a systematic investigation and correlation of crystalline and amorphous bifunctional electrocatalytic activity for NiFe oxides in alkaline media by the flame oxide‐synthesis method. They discovered that the amorphous NiFe oxide improves the OER kinetics and reported an overpotential of 340 mV at 10 mA cm^−2^, whereas crystalline NiFe oxide improves HER activity and reported an overpotential of 300 mV, −10 mA cm^−2^ [29]. Furthermore, Bak et al. showed that Fe doping, irrespective of the method, enhances OER activity in amorphous NiO films developed by the sol–gel method, contrary to crystalline NiO films, where the method of Fe substitution plays a critical role. In both crystalline and amorphous NiO, OER activity was enhanced, but the crystalline substrate exhibited excellent performance because of better Fe‐O coordination, hence emphasizing the importance of crystallinity and electronic structure along with chemical modifications [30]. Interestingly, Cai et al. conducted a comparative study of crystalline vs amorphous NiFe alloy by the room temperature solution method, finding that the OER activity for amorphous NiFe alloy with an atomic ratio of 3:1 exhibited superior performance than its crystalline counterpart owing to a higher density of active sites and greater structural flexibility [31]. Whereas, Li et al. developed amorphous NiFe‐based electrocatalysts via a hydrothermal method, where the crystallinity was deliberately suppressed through the synergistic templating action of CTAB and the citric acid‐sodium citrate buffer (pH 5) to achieve an amorphous matrix filled with pores, and the electrocatalyst with Ni to Fe ratio equal to 3:1 exhibited superior OER performance owing to enhanced exposure to electrocatalytically active sites [32]. Furthermore, Moschkowitsch et al. synthesized high surface area NiFe oxide aerogels (164–617 m^2^ g^−2^) via the modified epoxide route, achieving optimal OER performance at Ni:Fe of 94:6 despite modest surface area relative to other Fe‐richer compositions. They concluded that the metal ratio dominates activity over surface area, as excessive porosity induces mass transport limitations [33]. Subsequently, Lakhanlal et al. showed that heat treatment at 150°C of these aerogels removes crystalline interlayer water, further optimizing OER performance by destabilizing key intermediates [34]. This reinforces composition (Ni:Fe ratios) and post‐synthesis tuning for porosity/crystallinity in NiFe‐oxides play a key role in optimal OER performance. On the other hand, Mirizzi et al. conducted a thorough investigation of amorphous NiFe oxides with various molar ratios synthesized by the sol–gel method and reported superior performance for Ni_0.75_Fe_0.25_O [22]. Although Yu et al. observed maximum current densities for Ni:Fe ratio at 32:1 [35], Kumar et al. reported the best performance for ca. 200 nm NiFe oxide nanocubes with a molar ratio of 3:2, deriving from a composition made by *α*‐Fe_2_O_3_, *γ*‐Fe_2_O_3_, and NiO [36]. Furthermore, Zuber et al. demonstrated the impact of precursor materials for the synthesis of NiFe oxide on OER activity due to the occurrence of multiple phases. For this reason, the synthesis route also controls the structure and the electrocatalytic performance [37]. Recent studies reveal conflicting optimal Ni:Fe ratios (from 3:1 to 32:1) across synthesis methods, highlighting structure‐dependent activity: Amorphous NiFe oxides excel in OER kinetics, supported by the study of Shi et al. [29], whereas crystalline phases enhance stability via Fe‐O coordination as advocated by Bak et al. [30]. From the available literature, it can be concluded that for every synthetic route, the stoichiometries of the compounds (particularly the metal ratio) and the role of crystallinity in modulating the electrocatalytic activity of NiFe‐based oxides are imperative factors for OER performance and eventually lead to scalability of AEMWEs. Because amorphous NiFe‐based oxide electrocatalysts provide rich defective surfaces and wide grain boundaries for better OER kinetics, the benefits of crystalline NiFe oxide in terms of structural stability, better electronic conductivity, and operational durability cannot be subdued. Nowadays, modification in the crystallite size, geometrical orientation, phase purity, and lattice strain of NiFe oxides has emerged as a promising strategy to optimize intrinsic activity while maintaining mechanical robustness [23, 24]. Yet, no systematic investigation has evaluated these effects using fixed homogeneous precipitation or quantified transitions from amorphous to crystalline regimes. To address this knowledge gap, we have developed a simple, quick, and cost‐effective homogenous precipitation method to synthesize a range of amorphous and crystalline Ni_*x*_Fe_1‐*x*_O.

Following physicochemical characterization to study morphology, structure, and surface chemistry, finally, electrochemical characterizations were performed to optimize the electrocatalyst ink formulation. Different inks prepared using different solvent combinations, binder quantity, and ECs loading were evaluated to investigate their influence on the OER activity using linear sweep voltammetry (LSV) with a rotating disk electrode (RDE). The optimization process involved parameters such as drying time, binder content, and the overpotential at 10 mA cm^−2^. After identifying the optimal ink composition, the remaining electrochemical characterizations of the samples were subsequently conducted. The synthesized Ni_*x*_Fe_1‐*x*_O with different ratios were compared, and Ni_0.28_Fe_0.72_O and Ni_0.66_Fe_0.34_O showed overall superior performance as electrocatalysts in alkaline media with an average overpotential of ca. 359 mV and 359.1 mV, respectively. Furthermore, performance and stability tests of about 90 h were conducted for both samples in a laboratory‐scale full cell electrolyzer test.

## 2 Materials and Methods

### 2.1 Materials

All reagents were used as received without further purification. Potassium hydroxide (KOH, 85%, CAS 1310‐58‐3) was supplied by Sigma‐Aldrich. Iron(III) chloride anhydrous (FeCl_3_, 99.8%, CAS 7705‐08‐0) and aqueous ammonia (NH_3_, 25% w/w) were obtained from VWR Chemicals. Nickel(II) nitrate hexahydrate (Ni(NO_3_)_2_ · 6H_2_O, >98.5%, CAS 13 478‐00−7) and ethanol (CH_3_CH_2_OH, >99.8%, CAS 64‐17−5) were purchased from Honeywell, whereas 5% Nafion dispersion in water/ethanol was acquired from Alfa Aesar. Twenty wt% PiperION‐A TP‐85 and PiperION membrane (40 μm) were obtained from Versogen. Milli‐Q water with a resistivity of 18.2 MΩ·cm was used throughout.

### 2.2 Synthesis Procedure

**FIGURE 1:**
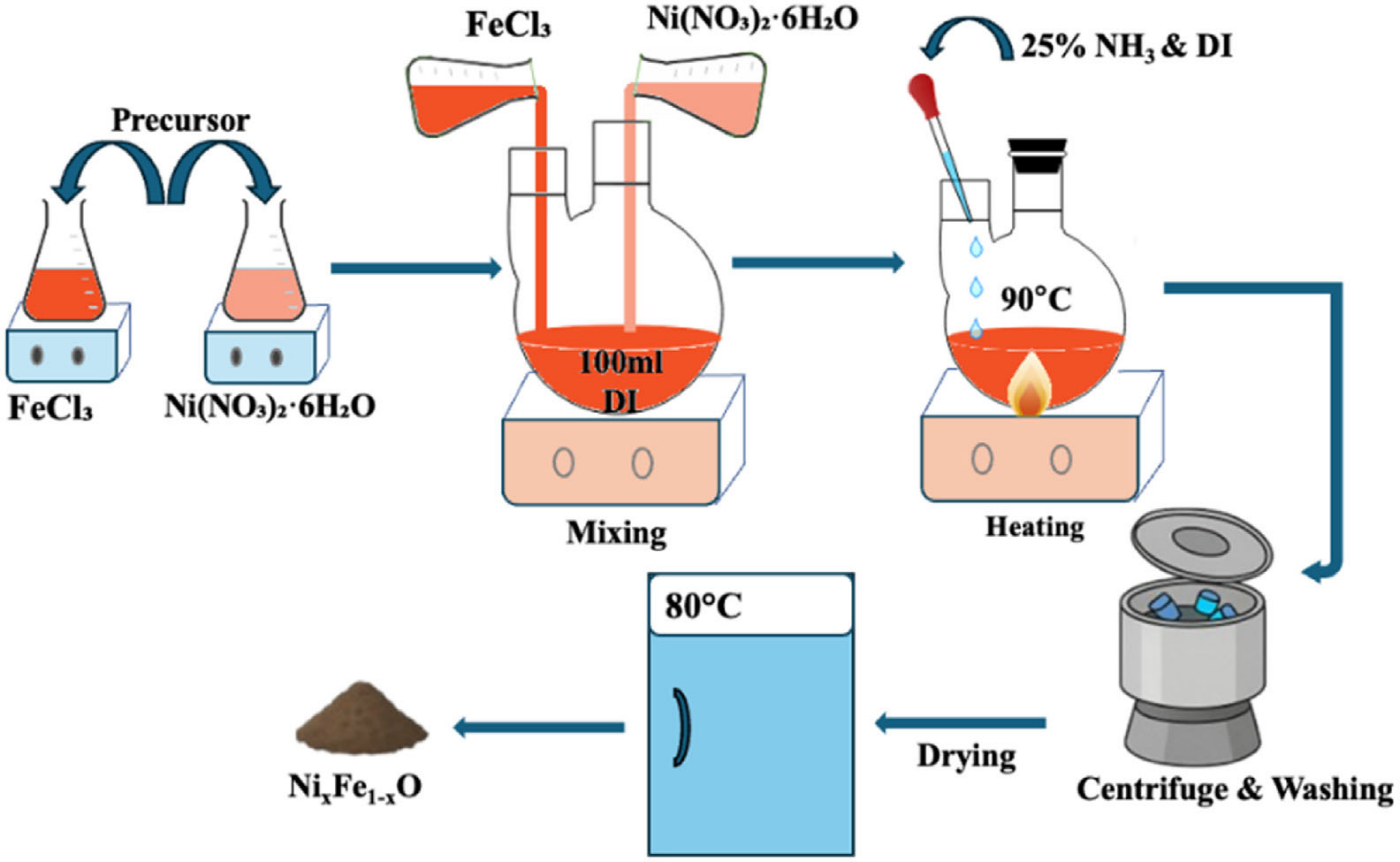
Schematic of the synthesis process for Ni_*x*_Fe_1‐x_O.

Iron(III) chloride (FeCl_3_) and nickel(II) nitrate hexahydrate (Ni(NO_3_)_2_ · 6H_2_O) were used as precursors to achieve 3 g of the final product. The precise quantity of precursors used is listed in Table 1. Precursors were dissolved in 100 mL of DI water while being stirred magnetically at room temperature for 5 min, then the mixture was heated to 90°C. After heating the mixture to 90°C, 5 mL of DI water and 10 mL of 25% ammonia aqueous solution were added. Afterward, the mixture was continuously stirred while being kept at 90°C for 30 min. Subsequently, the mixture was cooled down to room temperature. The product slurry was taken out, centrifuged, and washed three times with DI water and once with ethanol. Finally, the product was dried overnight at 80°C and was collected and ground for further analysis. Figure 1 shows the graphical representation of the synthesis route. For clarity, the notation Ni_*x*_Fe_1‐*x*_O is retained throughout the manuscript as a nominal sample code reflecting the targeted Ni:Fe composition. No post‐treatment was carried out to tune the crystallinity of the synthesized samples. Instead, phase distribution and crystallinity are primarily controlled by the Ni:Fe precursor ratio during the synthesis.

**TABLE 1:** Quantity of precursors for Ni_*x*_Fe_1‐*x*_O synthesis.

Description	Ni:Fe Molar Ratio	Ni(NO_3_)_2_ · 6H_2_O, g	FeCl_3_, g
Ni_0.28_Fe_0.72_O	28:72	4.17	6.17
Ni_0.35_Fe_0.65_O	35:65	4.76	4.44
Ni_0.66_Fe_0.34_O	66:34	7.00	2.10
Ni_0.85_Fe_0.15_O	85:15	8.58	0.89

### 2.3 Material Characterization

#### 2.3.1 X‐Ray Fluorescence (XRF) Instrument

X‐ray fluorescence (XRF) spectra were acquired using a Bruker Artax AXS instrument equipped with Mo source, operating over an energy range of 0–25 keV. For analysis, the spectra were restricted to the energy region of interest between 6 and 9 keV.

#### 2.3.2 X‐Ray Diffraction Instrument

A Rigaku Mini‐Flex 600 diffractometer equipped with a Cu K*α* (1.54 Å) radiation source operating at 40 kV and 15 mA was used to perform X‐ray diffraction (XRD). With an angular velocity of 2° per minute and a step size of 0.02°, the diffractograms were obtained between 10° and 80° 2*θ*. PDXL‐2 software was used to compare reference diffraction patterns from the JCPDS database.

#### 2.3.3 Scanning Electron Microscopy

The morphology and microstructure of the samples were examined by a desktop scanning electron microscopy (SEM) using a Fisher Phenom Pro G6, images were acquired at an accelerating voltage of 10.0 kV using the backscatter detector (BSD). Prior to analysis, powdered samples were covered with a conductive Au‐based coating by sputtering for 40 s using a VPI SD‐900C instrument to minimize charging effects.

#### 2.3.4 X‐Ray Photoelectron Spectroscopy Instrument

To gain deeper insight into the surface composition and electronic structure of the synthesized catalysts, X‐ray photoelectron spectroscopy (XPS) analyses were performed on the Ni_0.28_Fe_0.72_O and Ni_0.66_Fe_0.34_O samples. The measurements were conducted using a Physical Electronics ESCA System PHI 5800 spectrometer equipped with a monochromatic Al K*α* x‐ray source (hν = 1486.6 eV, 350 W). The binding energy scale was calibrated using the C 1s peak at 284.8 eV as an internal reference.

A comprehensive deconvolution of the Ni 2p, Fe 2p_3_/_2_, and O 1s core‐level spectra was carried out to determine the oxidation states and chemical environments of the surface species. The fitting procedure was performed using the MultiPak V6.1A software, whereas the deconvoluted peak areas were subsequently integrated and analyzed using MATLAB to obtain the relative atomic ratios and quantify the different oxidation states.

#### 2.3.5 High‐Resolution Transmission Electron Microscopy

HR‐TEM, scanning transmission electron microscopy (STEM), and energy‐dispersive x‐ray spectroscopy (EDX) were performed on a Thermo Fisher Talos F200X G2 microscope operated at 200 kV. EDX mapping was acquired using a SuperX system with four 30 mm^2^ silicon drift detectors (collection angle 0.7 sr). The datasets were processed and visualized using the Thermo Fisher VELOX software package.

#### 2.3.6 RDE for Electrochemical Measurements

In a typical three‐electrode glass cell (Pine), electrochemical measurements were performed using an Ag/AgCl/Cl‐(sat) electrode as the reference electrode, a platinum wire as the counter electrode, and a glassy carbon rotating disk electrode (*Ø* 5 mm, area of 0.1963 cm^2^) as the working electrode. The reversible hydrogen electrode (RHE) was then converted from the potential using the following formula:

$$E_{\left(\mathrm{RHE}\right)}=E_{\left(\mathrm{Ag/AgCl/}{\mathrm{Cl}}^{‐}\right)}+{E^{0}}_{\left(\mathrm{Ag/AgCl/}{\mathrm{Cl}}^{‐}\right)}+0.059\;pH$$

where *E* ^0^_(Ag/AgCl/Cl_^−^_)_  = 0.1976 V.

Linear sweep voltammograms (LSVs) were recorded in 1 M KOH between 1.2 and 1.9 V versus RHE at 5 mV s^−1^, whereas RDE was rotated at 1600 rpm. Prior to the acquisition of polarization curves, the catalyst layer was conditioned by cyclic voltammetry at a higher scan rate of 50 mV s^−1^ until a stable current was achieved. Electrolyte was purged with nitrogen gas to remove the dissolved oxygen from the solution at the beginning for 30 min, and a minute flow was maintained during the measurement to minimize bubble accumulation interference to allow the produced oxygen to escape from the solution. Tafel plots were extrapolated from the LSV measurements by plotting the overpotential (*η*) as a function of the logarithm of the current density. For Tafel plots all measured potentials (*η*′) were manually iR‐corrected for compensation of electrolytic resistance using the following equation:

$$\eta =\eta \prime -iR_{u}$$

where ***i*** is the electrode current and ***R*_u_** [Ω] is the uncompensated resistance extracted from the electrochemical impedance spectroscopy (EIS). The Ohmic drop (iR) correction was performed on the same electrochemical setup; however, for this purpose, a BioLogic SP‐150 potentiostat with a built‐in IR determining mode of the EC‐Lab software was used. After estimating the iR drop, 85% compensation was manually applied to the measured polarization curves. The electrochemical surface area (ECSA) was estimated from double‐layer capacitance ($C_{dl}$) measured by cyclic voltammetry (CV) in the non‐Faradaic region 0.175–0.275 V against Ag/AgCl. CVs were recorded at scan rates of 2, 10, 20 and 50 mV s^−1^. $C_{dl}$ was determined from the slope of Δj/2 (= $(j_{\text{anode}}-j_{\text{cathode}})/2$) versus scan rate. ECSA was calculated as follows:

$$ECSA=\frac{C_{dl}}{C_{s}}$$

where ***C***_**s**_ is the specific capacitance, and its value was taken as 40 µF cm^−2^ [38].

#### 2.3.7 Anion Exchange Membrane Water Electrolysis Test

The membrane electrode assemblies (MEAs) for electrochemical characterization in the Anion Exchange Membrane (AEM) water electrolyzer configuration were prepared using a spray‐coating technique. The Ni_0.28_Fe_0.72_O or Ni_0.66_Fe_0.34_O electrocatalysts were deposited on the anode side with a total loading of 4.5 mg cm^−2^. Half of the electrocatalyst was applied onto the Ni‐felt substrate (Bekipor ST Sintered Metal Fiber Matrix, type 2Ni06−020, Bekaert), and the other half onto the PiperION membrane (thickness 40 μm, Versogen). On the cathode side, a gas diffusion electrode (GDE) with a Pt loading (Pt/C) of 0.2 mg cm^−2^ was employed. In both electrodes, 20 wt% PiperION ionomer was used for the preparation of the catalytic inks.

Electrochemical measurements were performed in an electrolysis cell equipped with nickel plates featuring a single‐serpentine flow field (5 cm^2^) and gold‐plated current collectors. The tests were conducted at three different temperatures (40°C, 60°C, and 80°C), feeding the anode side with an alkaline aqueous solution (1 M KOH) at a flow rate of 5 mL min^−1^. LSV measurements were carried out using an IVIUM Technologies XP 40 potentiostat–galvanostat, with LSV curves recorded at a scan rate of 5 mV s^−1^. Chronopotentiometric and EIS analyses were conducted using an Autolab PGSTAT302N potentiostat–galvanostat equipped with a Frequency Response Analyzer (FRA) module (Metrohm). EIS measurements were performed under potentiostatic control at a cell voltage of 1.8 V over a frequency range from 10 kHz to 100 mHz, employing a single‐sine frequency sweep mode. The amplitude of the sinusoidal perturbation was set to 0.01 V. The series resistance (*R*_s_) was determined from the high‐frequency intercept on the real axis of the Nyquist plot. A short‐term stability test (ca. 90 h) was performed at 80°C under a constant current density of 1 A cm^−2^ to evaluate the durability of the system.

## 3 Results and Discussion

### 3.1 Physicochemical Characterizations

**FIGURE 2:**
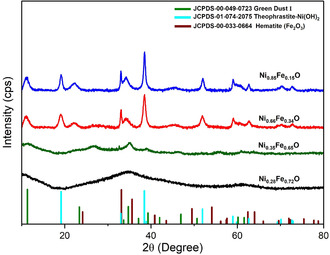
XRD Diffractograms of Ni_*x*_Fe_1‐*x*_O electrocatalysts. Legend: Ni_0.28_Fe_0.72_O (black line), Ni_0.35_Fe_0.65_O (green line), Ni_0.66_Fe_0.34_O (red line) and Ni_0.85_Fe_0.15_O (blue line). Reference patterns: Green dust [FeNiCl(OH)_4_ x H_2_O] (green), Theophrastite Ni(OH)_2_ (cyan), and Hematite α‐Fe_2_O_3_ (brown).

The elemental composition of the various NiFe oxides (with different Ni:Fe ratios) was analyzed via x‐ray fluorescence spectroscopy (XRF). The XRF spectra of the Ni_*x*_Fe_1‐*x*_O samples in Figure S1 exhibit characteristic Fe K*α* (6.4 keV) and Ni K*α* (7.48 keV) peaks, confirming the coexistence of both elements in all compositions. The relative intensity of the Fe K*α* peak increases with higher Fe loading, consistent with the intended stoichiometric variation. Afterward, the crystalline structure and crystallite size were then examined by XRD diffraction, and the corresponding patterns are reported in Figure 2, which clearly illustrates how crystallinity evolves by increasing the Ni %_mol_.

Across the samples, distinct peaks at 2*θ* = 11.3° (003), 33.7° (100), 34.7° (102), and 60.1° (110) are consistent with the presence of layered hydroxide/LDH‐like domains (card number 049–0723, ICDD). Because chloride‐containing precursors were used during synthesis, these layered domains may also involve chloride‐containing hydroxide environments or chloride‐intercalated layered structures (e.g., [FeNiCl(OH)_4_ x H_2_O) [39, 40]. On the other hand, the obtention of Ni(OH)_2_ was corroborated with the presence of the peaks at 2*θ* = 19.1° (001), 33.0° (100), 38.4° (011), 51.9° (012), 59.0° (110), 62.6° (111), 70.1° (103), and 72.9° (112) (card number 01‐074−2075, ICDD), which, according with the literature, this structure phase belongs to the layered double hydroxide (LDH) phase and possibly NiFe LDH structures [41, 42]. The presence of some peak signals at 2*θ* = 33.2° (104), 64.0° (300), 56.2° (211), 69.6° (208), belonging to α‐Fe_2_O_3_ (card number 033–0664, ICDD). However, since several of these reflections overlap with those of Ni(OH)_2_‐related phases, and LDH‐like hydroxides typically exhibit broad peaks due to small coherent domain size and the partial amorphous character of the samples, an unambiguous phase assignment based solely on the XRD analysis remains challenging. Therefore, the notation Ni_*x*_Fe_1‐*x*_O is used here only as a nominal compositional descriptor rather than as evidence of a single crystallographic phase. Moreover, the XRD patterns clearly show that the relative phase contribution and crystallinity strongly depend on the Ni:Fe ratio. In particular, the Ni_0.28_Fe_0.72_O, sample exhibits broad and weak reflections, thus indicating mostly an amorphous structure with a poorly ordered structure with limited long‐range order [43]. Whereas, by increasing the Ni %_mol_, diffraction peaks become progressively sharper and more intense, revealing a gradual increase in crystallinity. Together, these results reveal a phase mixture in which the relative crystallinity and phase contributions depend strongly on the Ni:Fe ratio. These observations suggest that the structural differences shown in Figure S2 arise from changes in the relative proportion and ordering of hydroxide/LDH‐like and Fe‐containing domains formed during the synthesis process.

**FIGURE 3:**
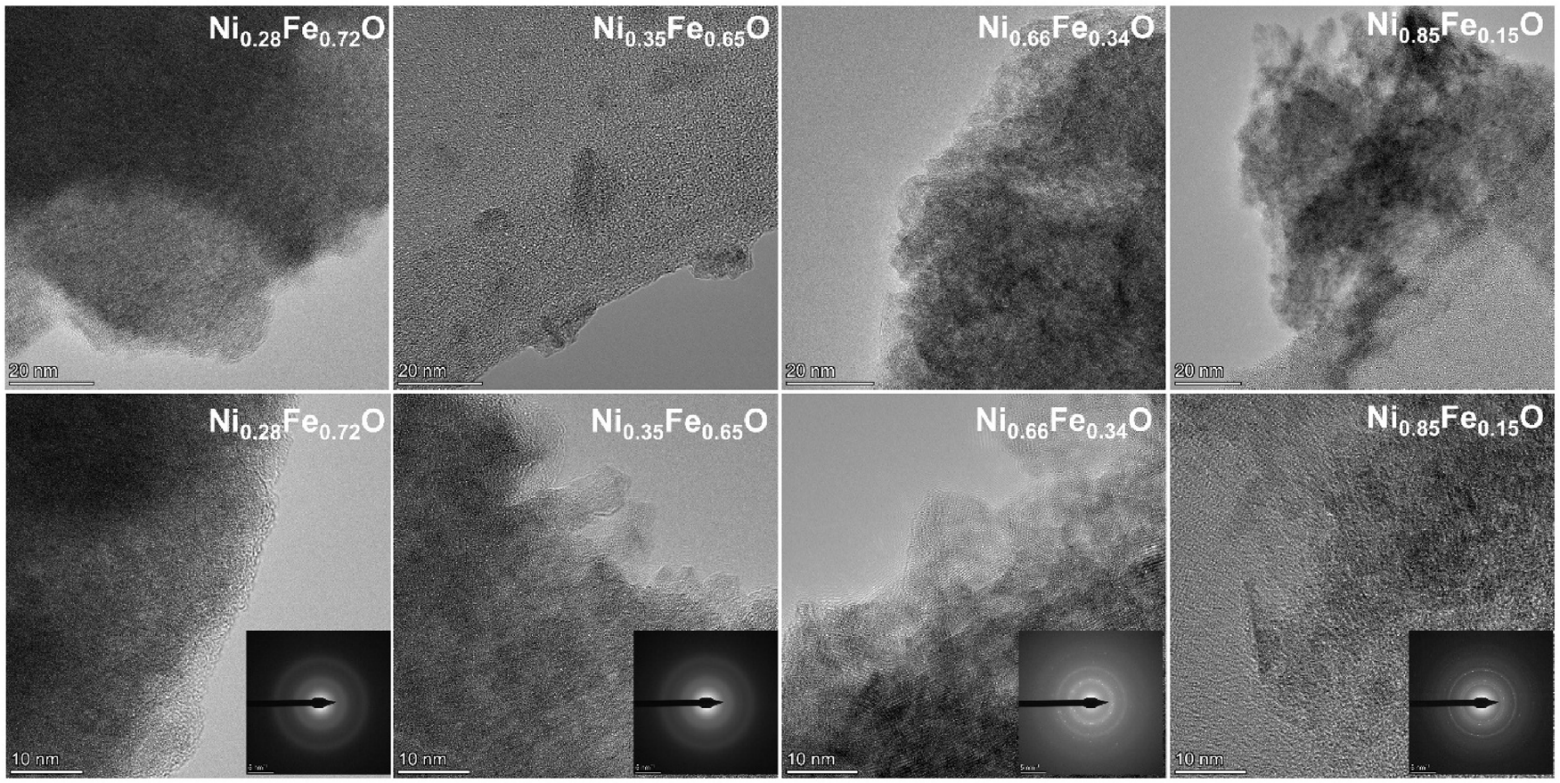
HR‐TEM images at low (top) and high (bottom) magnification of the synthesized samples following the order of Ni %_mol_ from left to right: Ni_0.28_Fe_0.72_O, Ni_0.35_Fe_0.65_O, Ni_0.66_Fe_0.34_O, and Ni_0.85_Fe_0.15_O.

Furthermore, the morphological characteristics of the synthesized Ni_*x*_Fe_1‐*x*_O samples were initially investigated by SEM, revealing distinct morphological differences as shown in Figure S2(a–d). The sample Ni_0.28_Fe_0.72_O with the lowest Ni content exhibited a predominantly agglomerated and irregular morphology. In contrast, Ni‐rich compositions like Ni_0.35_Fe_0.65_O, Ni_0.66_Fe_0.34_O, and Ni_0.85_Fe_0.15_O, displayed a denser, plate‐like morphology with larger particles. These structural variations suggest that the Ni:Fe ratio significantly influences nucleation and crystal growth. Additionally, to gain a profound understanding of their microstructure and elemental distribution, HR‐TEM coupled with energy‐dispersive x‐ray spectroscopy (EDX) was performed. The HR‐TEM images of the Ni_*x*_Fe_1‐*x*_O in Figure 3 indicate that Ni_0.28_Fe_0.72_O shows mainly an amorphous structure with a diffuse SAED pattern and faint lattice fringes, suggestive of small, disordered domains; hence, poor crystallinity and clear SAED patterns were obtained for Ni_0.66_Fe_0.34_O and Ni_0.85_Fe_0.15_O already, as shown in Figure 3 and Figure S3. Furthermore, almost all samples exhibit lattice fringes with d‐spacings of 0.25–0.26 nm (corresponding to the reflections at approximately 2*θ* =34°‐36°), and 0.15 nm (associated with the reflection peaks at about 2*θ* = 60°‐64°), consistently matching the three phases identified by XRD. The most Ni‐rich sample Ni_0.85_Fe_0.15_O additionally shows a d‐spacing of 0.23 nm (2*θ* = 38.4°), which can be assigned to Ni(OH)_2._

**FIGURE 4:**
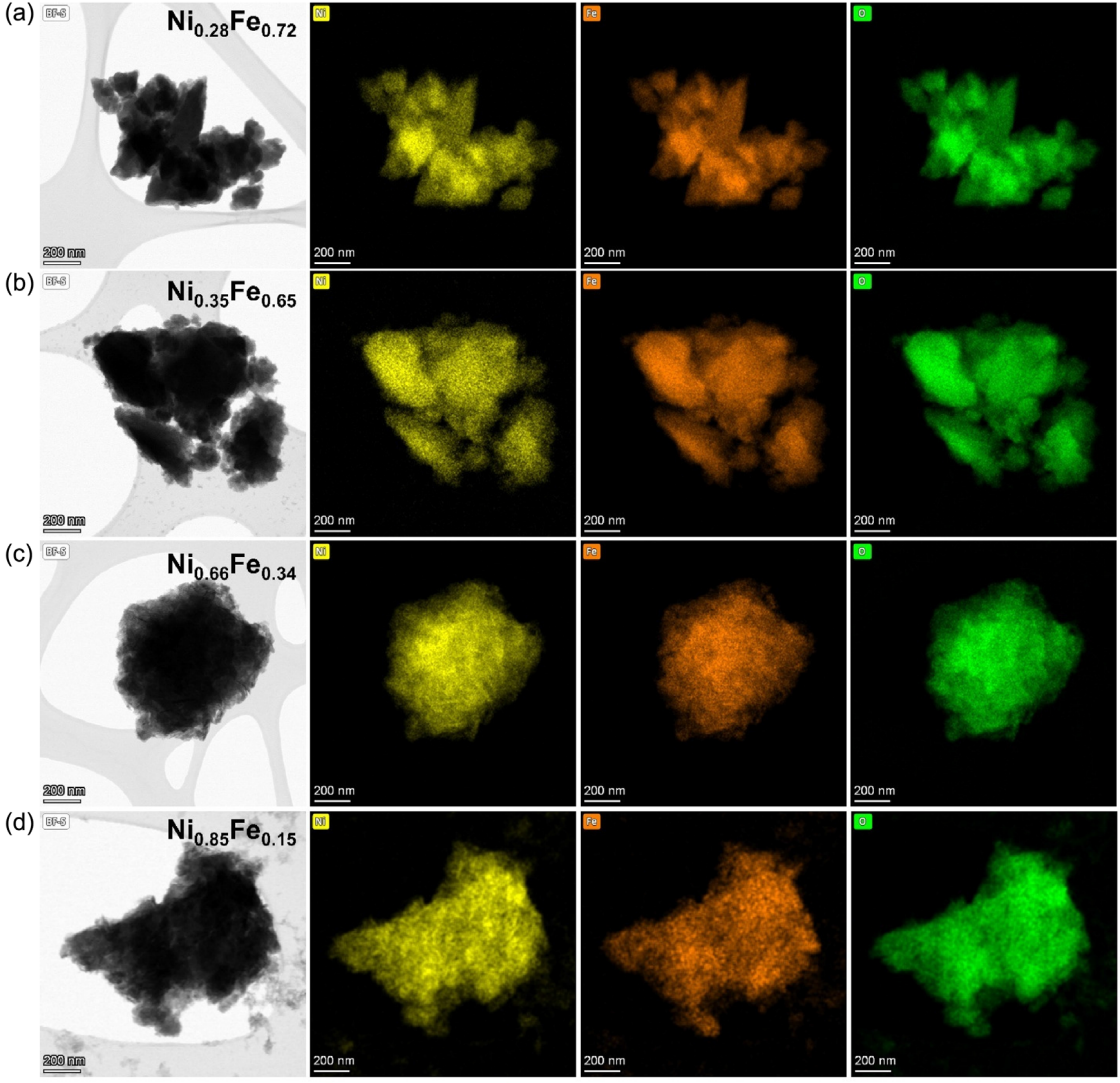
STEM images of samples (a) Ni_0.28_Fe_0.72_O, (b) Ni_0.35_Fe_0.65_O, (c) Ni_0.66_Fe_0.34_O, (d) Ni_0.85_Fe_0.15_O, from left to right columns: Bright field images, Ni map, Fe Map, O map.

Moreover, EDX was performed at different regions of the samples to evaluate the elemental distribution in the Ni_*x*_Fe_1‐*x*_O, as shown in Figure 4. The elemental maps clearly show the homogenous distribution of Fe and Ni in all the samples except for Ni_0.85_Fe_0.15_O, which showed poorly distributed Fe particles. Figure S4 shows a comparison between Ni_0.28_Fe_0.72_O and Ni_0.85_Fe_0.15_O, showing the presence of Ni‐rich phases only in Ni_0.85_Fe_0.15_O, which exhibits a more powder‐like morphology compared with the more aggregated nature of the other samples. This distinct morphology is consistent with the more pronounced crystalline character of the Ni‐rich sample relative to the others. In addition, both Ni_0.35_Fe_0.65_O and Ni_0.66_Fe_0.34_O contain less than 5%_at._ Cl as impurity from unreacted precursors, probably in the form of green dust [FeNiCl(OH)_4_xH_2_O], in agreement with the XRD analysis. A comparative bar chart of the theoretical Fe/Fe+Ni ratios expected from the synthesis stoichiometry with the actual elemental ratios measured by EDX for Ni_*x*_Fe_1‐*x*_O electrocatalysts is given in Figure S5. Overall, the measured values show good agreement with theoretical values, with minor exceptions noted for Ni_0.85_Fe_0.15_O and Ni_0.35_Fe_0.65_O. Where Ni_0.85_Fe_0.15_O shows a Fe content of 28% instead of 15%, whereas Ni_0.35_Fe_0.65_O shows a Fe content of 78% instead of 65%. These deviations likely reflect the multiphase nature of the materials, local compositional heterogeneity, and the fact that EDX probes selected regions rather than the bulk average composition.

X‐ray photoelectron spectroscopy (XPS) was used to investigate the surface chemical composition and oxidation states of the synthesized Ni_0.28_Fe_0.72_O and Ni_0.66_Fe_0.34_O electrocatalysts in detail. The selected samples were analyzed to assess and compare the surface chemistry of an amorphous Ni_0.28_Fe_0.72_O and a crystalline Ni_0.66_Fe_0.34_O species.

**FIGURE 5:**
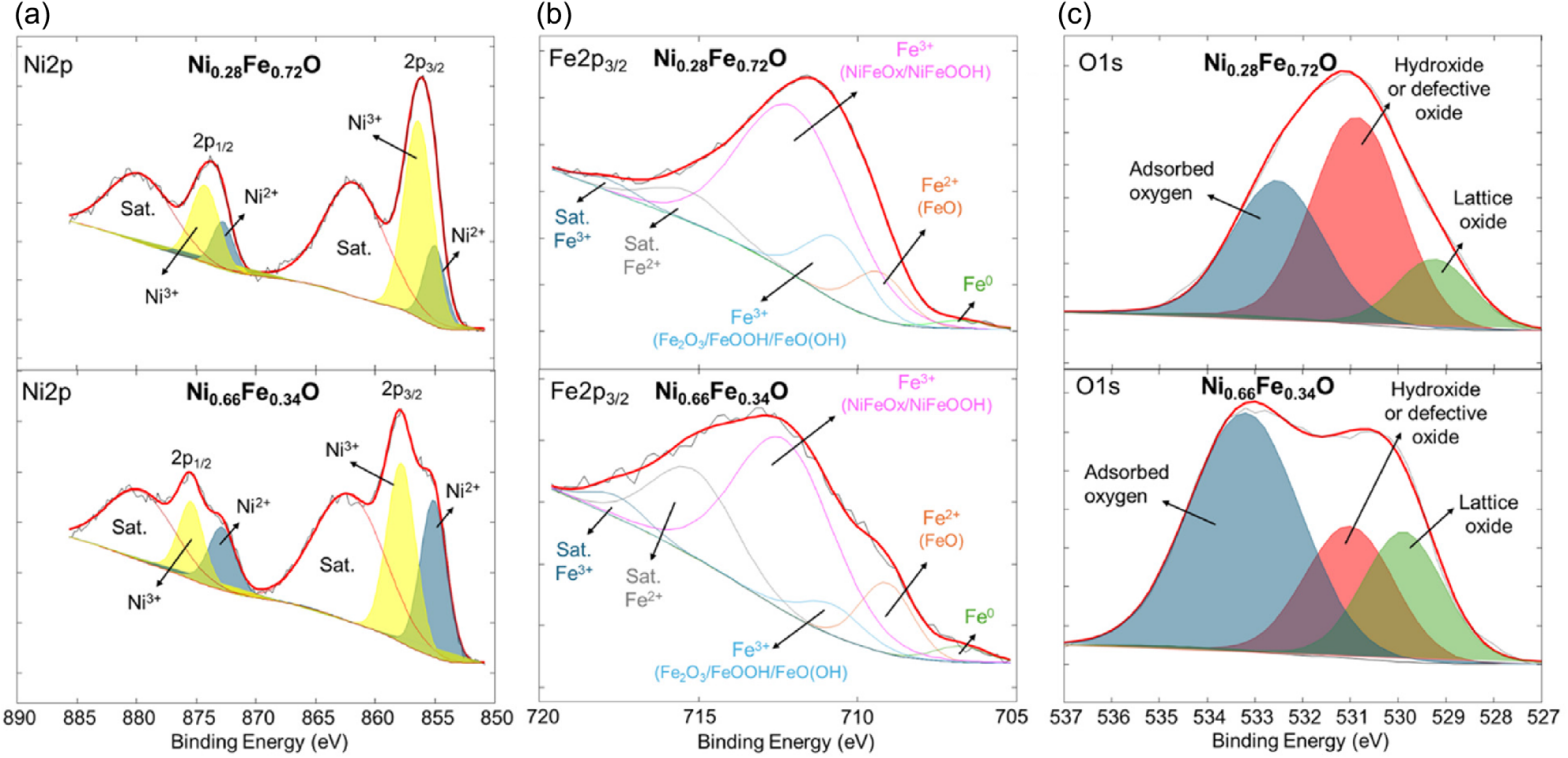
XPS deconvolution of (a) Ni2p, (b) Fe2p_3/2,_ and (c) O1s for Ni_0.28_Fe_0.72_O and Ni_0.66_Fe_0.34_O.

The Ni 2p spectra (Figure 5a) exhibit the characteristic features of Ni^2+^ and Ni^3+^ species, with no detectable signals attributable to metallic Ni^0^, generally found at binding energies ranging between 852.4 and 852.8 eV. The main peaks associated with Ni^2+^ appear at 855.0 eV (2p_3/2_) and 872.9 eV (2p_1/2_), whereas Ni^3+^ contributes components at 856.4 eV and 874.3 eV [22, 44, 45]. Two intense shake‐up satellites located at 861.7 eV and 879.6 eV further confirm the oxidized nature of the nickel species [46, 47]. The Ni^3+^/Ni^2+^ ratio decreases by more than a factor of two, from 2.5 in Ni_0.28_Fe_0.72_O to 1.2 in Ni_0.66_Fe_0.34_O, indicating a higher relative abundance of Ni(III) species in the more amorphous sample. Conversely, the Ni(III) peak in Ni_0.66_Fe_0.34_O is slightly shifted toward higher binding energies, suggesting the predominance of more strongly oxidized species such as NiOOH, rather than Ni_2_O_3_, or possibly the formation of FeNiCl(OH)_4_‐type surface complexes. The Fe 2p_3/2_ spectra of the Ni_0.28_Fe_0.72_O and Ni_0.66_Fe_0.34_O electrocatalysts are presented in Figure 5b. The signal at 706.8 eV corresponds to metallic Fe (Fe^0^), which is not accompanied by a pronounced satellite feature. The Fe^2+^ species, typical of FeO‐like coordination, appears at 709.2 eV and is associated with a satellite approximately 6 eV higher in binding energy. The Fe^3+^ components, ascribed to Fe_2_O_3_, FeOOH, or FeO(OH) phases, as well as Fe^3+^ species within NiFeO_*x*_ or NiFeOOH, are observed at 710.7 eV and 712.0 eV, respectively. The characteristic Fe^3+^ satellite lies about 7–8 eV above the main Fe 2p_3/2_ peak. Both samples exhibit a dominant contribution from Fe^3+^ species in NiFeO_*x*_/NiFeOOH environments, with the Ni_0.66_Fe_0.34_O sample showing a slight shift toward higher binding energy, suggesting a more oxidized surface state. The O 1s spectra (Figure 5c) display three main contributions at 529.3 eV, 530.9 eV, and 532.5 eV, attributed respectively to lattice oxygen (O^2‐^), hydroxyl or defect‐related oxygen (M‐OH), and surface‐adsorbed species [48, 49]. Upon fitting, the relative intensity of the lattice oxygen peak increases with Fe content in the NiFeO_*x*_ materials, consistent with a structural evolution from an amorphous network to a more ordered oxide lattice, as corroborated by the XRD results.

### 3.2 Electrochemical Characterizations

#### 3.2.1 Optimization of the Ink Formulation in the RDE Experiments

**FIGURE 6:**
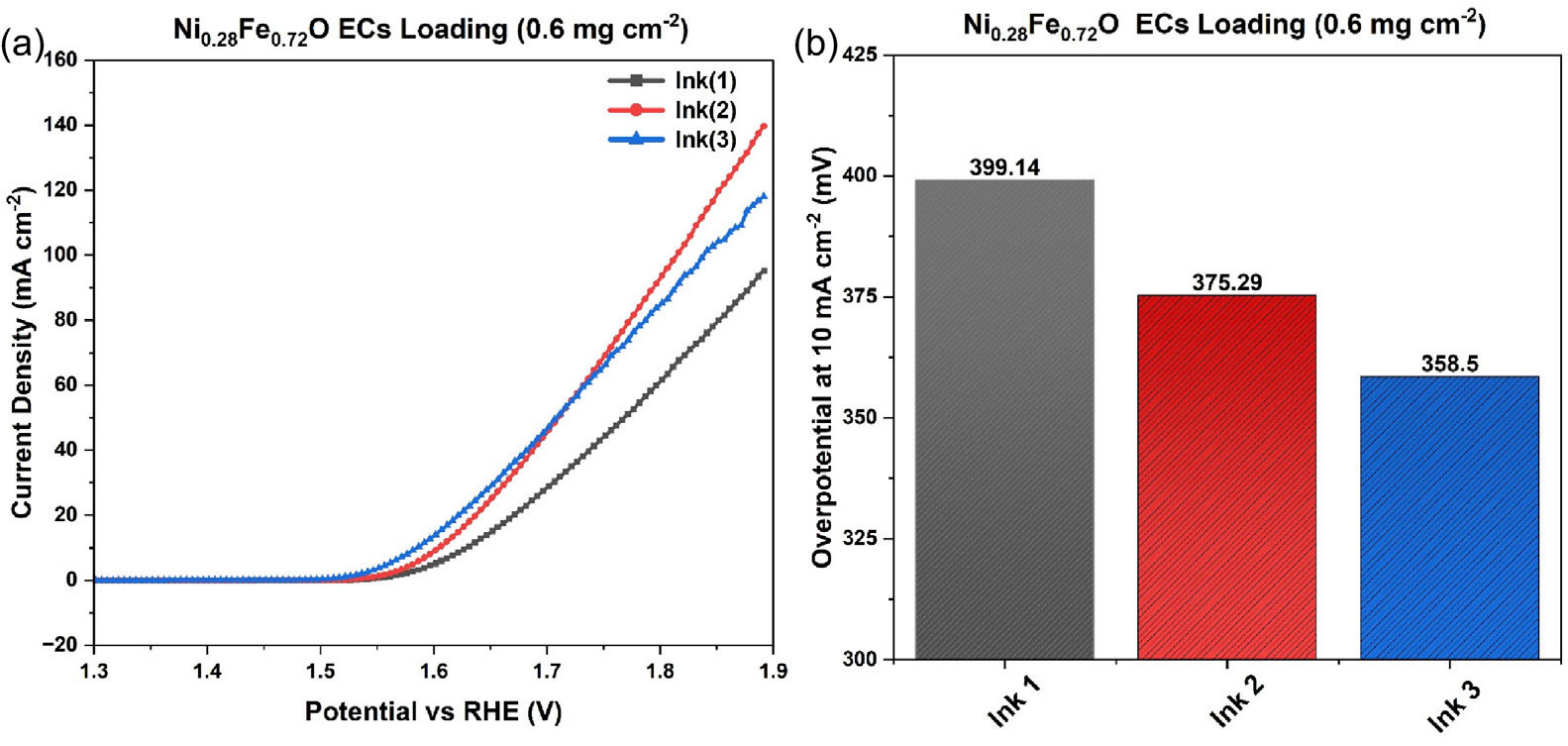
(a) Polarization curves for Ni_0.28_Fe_0.72_O and (b) Overpotentials measured at 10 mA cm^−2^.

Electrochemical performance of the synthesized Ni_*x*_Fe_1‐*x*_O was examined through RDE in the alkaline media using 1 M KOH (pH 14). Different ink recipes were used to investigate the effect of the ink composition on the OER electrocatalytic activity. The final objective was to evaluate an appropriate ink recipe that might influence the electrocatalytic performance during RDE experiments. Ni_0.28_Fe_0.72_O was used as ECs in ink formulation, as reported in Table 2. Ni_0.28_Fe_0.72_O with a loading of 0.6 mg cm^−2^ was investigated for comparison of the inks by using LSV. In Figure 6a, the LSVs of Ni_0.28_Fe_0.72_O are shown, whereas in Figure 6b, the overpotentials at a current density of 10 mA cm^−2^ are stated for Ni_0.28_Fe_0.72_O, revealing clear differences in the polarization curves, which are attributed to the variations in ink composition. Ink 1 was able to achieve a maximum current density of 95.3 mA cm^−2^ at the cost of a very high overpotential (399 mV at 10 mA cm^−2^). Whereas ink 2 showed a higher current density and lower overpotential, 139.72 and 375.29 mV at 10 mA cm^−2^, respectively, but due to the higher water content in the ink formulation, it significantly extended the drying time. However, for ink 3, the current density achieved for the same potential window was 118 mA cm^−2^ with the lowest overpotentials of 358.5 mV at 10 mA cm^−2^. The reduced water content in ink 3 also reduced the drying time, contributing to improved electrode performance. After comparative analysis and consideration of the performance parameters like drying time, quantity of binder, and overpotentials at 10 mA cm^−2^, ink 3 was unanimously selected to carry out the remaining electrochemical characterization of already synthesized samples.

**TABLE 2:** Different ink recipes used to investigate the influence of ink additives on OER activity.

Compound	Ink 1	Ink 2	Ink 3
ECs	5 mg	4 mg	5 mg
Isopropyl alcohol	985 µL	200 µL	785 µL
Water	/	768 µL	200 µL
Nafion solution, 5% in water/ethanol	15 µL	32 µL	15 µL

#### 3.2.2 Electrochemical Characterization with Different Electrocatalyst Loading in the RDE Experiments

**FIGURE 7:**
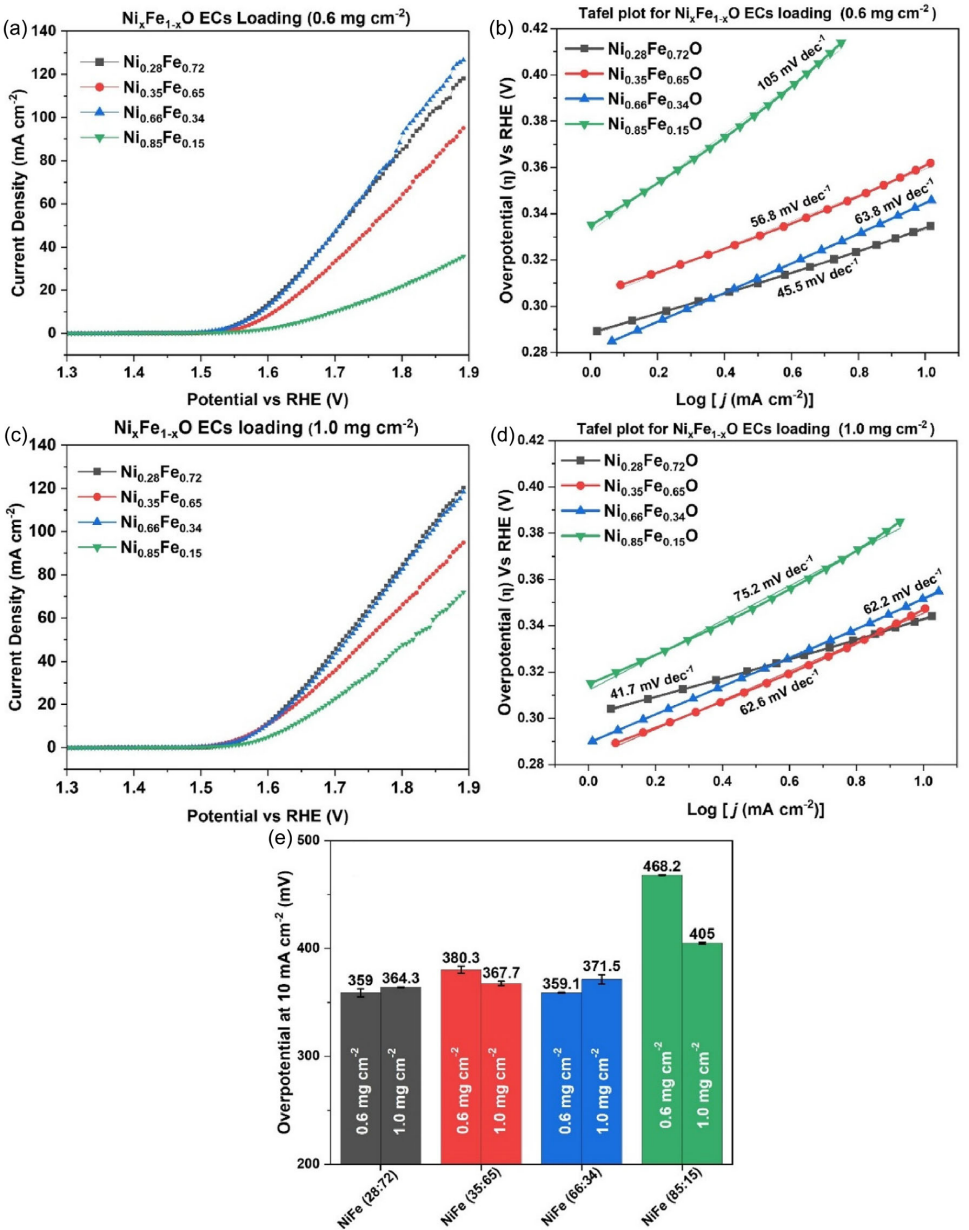
(a) Polarization curves of various ECs loading 0.6 mg cm^−2^ (b) Tafel plots at ECs loading 0.6 mg cm^−2^ (c) Polarization curves of various ECs loading 1.0 mg cm^−2^ (d) Tafel plots at ECs loading of 1.0 mg cm^−2^ (e) Mean overpotentials (uncompensated) measured at 10 mA cm^−2^ from different LSVs.

After analyzing the optimal ink formulation, polarization curves were obtained at two different ECs loading, that is, with 0.6 mg cm^−2^ and 1.0 mg cm^−2^, to examine the effect of ECs loading onto the glassy carbon electrode in RDE. Polarization curves at different loadings for all the synthesized samples are reported in Figure 7a‐c, whereas Figure 7b and Figure 7d show the Tafel slopes at different electrocatalyst loadings. Mean overpotentials from triplicate LSVs (non‐iR corrected) are reported in Figure 7e. Interestingly, catalyst loading effects are composition‐dependent. For instance, in the case of Fe‐rich ECs Ni_0.28_Fe_0.72_O, with the increase of electrocatalyst loading, the overpotential at 10 mA cm^−2^ is also increased, likely due to mass transport limitation and amorphous nature. Whereas Ni_0.35_Fe_0.65_O and Ni_0.85_Fe_0.15_O showed a decrease in overpotential at 10 mA cm^−2^ with an increase in the catalyst loading, correlated to better electronic conductivity due to crystallinity. Conversely, Ni_0.66_Fe_0.34_O overpotential at 10 mA cm^−2^ slightly increases with mass loading. However, Tafel slopes from Figure 7b and d reveal some loading‐dependent kinetics for Ni‐rich samples. For instance, at 0.6 mg cm^−2^, Ni_0.85_Fe_0.15_O reaches 105 mV dec^−1^, but at 1.0 mg cm^−2^, the value decreased to 75.2 mV dec^−1^. In the case of Ni_0.66_Fe_0.34_O, the effect is not that pronounced, but there is a minor decrease from 63.8 to 62.2 mV dec^−1^. Tafel analysis also provided some insightful information about reaction kinetics. Fe‐rich ECs Ni_0.28_Fe_0.72_O and Ni_0.35_Fe_0.65_O exhibited at 0.6 mg cm^−2^ a Tafel slope of 45.5 and 56.8 mV dec^−1^_,_ respectively, thus suggesting fast electron transfer and efficient reaction kinetics. Although Ni‐rich samples, Ni_0.85_Fe_0.15_O and Ni_0.66_Fe_0.34_O, showed a moderate Tafel slope of 105 and 63.8 mV dec^−1^, indicating that Fe‐rich phases lower the OER energy barrier. Furthermore, the ECSA of the synthesized Ni_*x*_Fe_1‐*x*_O electrocatalysts was estimated from the double‐layer capacitance (*C*_dl_) reported in Figure S6e. *C*_dl_ values range between 0.245 and 0.299 mF cm^−2^, suggestive of moderate ECSA typical of a thin film on GCE [50, 51]. As expected, Ni_0.28_Fe_0.72_O achieved the highest ECSA (*C*_dl_ = 0.299 mF cm^−2^), correlating with superior kinetics (45.5 mV dec^−1^). Therefore, among the synthesized electrocatalysts, the amorphous Ni_0.28_Fe_0.72_O with higher Fe content and the crystalline Ni_0.66_Fe_0.34_O with an optimal Ni:Fe ratio exhibited the most promising overall OER performance, achieving a current density of 118 and 126 mA cm^−2^ with a mean overpotential of 359 mV and 359.1 mV at 10 mA cm^−2^, polarization curves and replicate data are presented in (Figure S7 and Table S1), respectively. This superior performance can be attributed to the possible presence of active Ni(Fe)OOH sites formed due to the presence of proton‐deficient species of Ni^3+^ (NiOOH) [52, 53].

### 3.3 AEMWE Integration and Results

**FIGURE 8:**
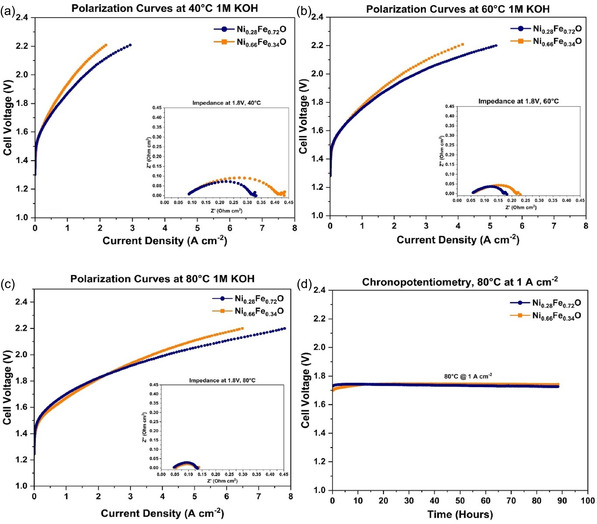
(a) LSVs at 40°C 1 M KOH of both samples along with EIS Nyquist plot at 1.8V as inset (b) LSVs at 60°C 1 M KOH of both samples along with EIS Nyquist plot at 1.8V as inset (c) LSVs at 80°C 1 M KOH of both samples along with EIS Nyquist plot at 1.8V as inset (d) Chrono potentiometric curve at 80°C, 1 M KOH solution and 1 A cm^−2^.

To transform the promising half‐cell OER activity into device‐level performance, Ni_0.28_Fe_0.72_O and Ni_0.66_Fe_0.34_O electrocatalysts were utilized as the anode material in a 5 cm^2^ single cell AEMWE. The cell incorporated PiperION membrane and ionomer on both electrodes, and polarization curves were acquired at 40°C, 60°C, and 80°C to evaluate the effect of temperature on the performance. The results in Figure 8a–c show that increasing the temperature results in improved performance. The highest performance achieved for Ni_0.28_Fe_0.72_O was at 80°C, delivering a current density of 7.81 A cm^−2^ against a cell voltage of 2.2 V and 4.14 A cm^−2^ at a cell voltage of 2.0 V. Whereas, Ni_0.66_Fe_0.34_O achieved 6.49 A cm^−2^ at 2.2 V and 3.69 A cm^−2^ at 2.0 V at 80°C. This improvement can be attributed to the higher ionic conductivity of the AEM, and the enhanced reaction kinetics at the electrode interfaces at higher temperatures, which can be further supported by the Nyquist plots (insets). Figure 8a–c recorded at 1.8 V, which shows a clear reduction in both the charge transfer resistance (*R*_ct_) and series resistance (*R*_s_). Details are reported in Table S1. A direct comparison with previously reported studies is challenging because experimental conditions such as the choice of AEM, electrolyte concentration, and electrocatalyst composition or loading vary widely across the literature. Nevertheless, to the best of our knowledge, the results obtained are best amongst Ni_*x*_Fe_*x*_O‐based electrocatalysts, even those employing higher electrocatalyst loadings and more optimized configurations, highlighting the competitive performance of the electrocatalysts. A comprehensive summary of recent studies investigating AEM water electrolyzers equipped with nonprecious anode electrocatalysts under 1 M KOH feed operated at 60°C is summarized in Table 3. Furthermore, a short‐term durability test (ca. 90 h) was carried out under galvanostatic conditions at 1 A cm^−2^, and the corresponding chronopotentiometry curves are shown in Figure 8d. Both Ni_0.28_Fe_0.72_O and Ni_0.66_Fe_0.34_O anodes exhibited a brief initial rise in cell potential, consistent with the expected activation phase and stabilization of the catalyst electrolyte interface. After this short transient, the cell voltage remained highly stable at approximately 1.74 V for the entire duration of the test, indicating excellent operational durability of both electrocatalysts when used as AEMWE anodes. Following the stability test, the cells were disassembled, and the catalyst layers mechanically removed from the Ni‐felt were examined by STEM‐EDS. Compared with the pristine materials, clear structural and compositional changes were shown in Figure S8 and Figure S9. Nickel and iron appeared largely segregated, with the formation of localized Fe‐oxide pillars and a fine Ni‐rich particulate phase. These features were more pronounced in the Ni_0.28_Fe_0.72_O sample than in Ni_0.66_Fe_0.34_O, and confirm that despite the excellent electrochemical stability recorded during operation, the catalysts do undergo structural and surface transformations. Such changes make it difficult to clearly identify the mechanisms responsible for enhanced performance, and no single explanation can yet be established. Nevertheless, it is evident that the degree of structural order plays a significant role and must be considered when designing highly active catalysts for the OER.

**TABLE 3:** Recent literature on AEMWEs employing various anode electrocatalysts operated at 60°C with 1 M KOH feed at the anode.

**MEA Configuration** **(Anode//Membrane//Cathode)**	**Anode mass loading**, **mg cm^−2^**	**Current density**, **A cm^−2^ @ 1.8 V**	**Current density**, **A cm^−2^** **@ 2.0 V**	**Current density**, **A cm^−2^ @ 2.2 V**	Ref.
**Ni**_**0.28**_ **Fe**_**0.72**_ **O//PiperION//Pt/C**	**4.5**	**1.24**	**2.69**	**5.19**	**This article**
**Ni**_**0.66**_ **Fe**_**0.34**_ **O//PiperION//Pt/C**	**4.5**	**1.12**	**2.26**	**4.14**	
(NiCo)_3_Se_4_/Ni foam//Sustainion x37‐50//Pt‐C/Carbon paper	2.0	—	2.0	—	[54]
Ni_0_._75_Fe_0_._25_O//PiperION//Pt/C	2.0	1.4	2.6	—	[22]
NiFe_2_O_4_/FAA3‐50 membrane//Pt/C	3.0	1.2	2.0	2.7	[55]
Ni foam//PiperION//Pt/C	—	0.38	0.87	—	[56]
Ni‐felt//Fumion Recast//Pt/C	—	0.73	1.3	2.05	[57]
Ni foam//PBP//Pt/C	—	0.56	1.25	2.8	[56]
NiFe_2_O_4_//Sustainion X37‐50//Ni Raney	2.0	0.74	—	—	[58]
NiFe_2_O_4_//FAS‐50//NiFeCo	2.0	0.28	0.92		[58]
NiAl/Porous SS//HMT‐PMBI AEM//NiAlMo/Porous SS	—	0.4	0.8	1.2	[59]
Ni–Fe//PTFE‐Sustainion//Ni–Fe	20	—	0.62	—	[60]
IrO_2_//PTFE‐Sustainion//Pt/C	2.0	1.0	1.8	—	[60]
NiFe_2_O_4_//A‐201//NiFeCo	—	0.18	0.96	2	[61]
CoCrO_*x*_//PiperION‐A60‐HCO3//Pt/C	1.0	0.9	1.2	1.5 @ 2.1V	[62]
CoSb_2_O_6_ /Ni foam//FAA‐3‐50,//Pt‐C	—	∼ 0.52	0.8 @1.9 V	—	[63]

## 4 Conclusions

In this study, NiFe‐based oxide electrocatalysts were synthesized via a homogeneous precipitation method and evaluated for the OER in AEMWE. Homogeneous precipitation reveals how composition controls crystallinity, with Fe‐rich compositions forming amorphous (oxy)hydroxides, whereas Ni‐dominant compositions yield crystalline phases. Overall, the combined XRD and EDX results demonstrate that the Ni:Fe ratio governs not only the nominal composition of the materials but also their degree of crystallinity, local elemental distribution, and multiphase character, which must all be considered when interpreting their physicochemical behavior. Hence, by tuning the Ni:Fe ratio, catalysts with distinct compositions and crystallinity can be engineered to achieve comparable OER performances, as evidenced by low values of mean overpotentials such as 359 mV at 10 mA cm^−2^ for Ni_0.28_Fe_0.72_O and 359.1 mV for Ni_0.66_Fe_0.34_O at a loading of 0.6 mg cm^−2^. The observed catalytic performance can be reasonably attributed to the synergistic interaction between Ni and Fe species, where Ni(OH)_2_ provides a conductive and structurally stabilizing matrix, while Fe contributes the primary OER‐active sites. Subsequent evaluation in a lab‐scale AEMWE device also demonstrated good electrolyzer performance and operational stability for both catalysts. Notably, a difference in the evolution of the operating potential was observed during prolonged operation: The amorphous Ni_0.28_Fe_0.72_O exhibited a slight decrease in potential over time, whereas the crystalline Ni_0.66_Fe_0.34_O showed a modest increase. While the underlying mechanisms require further investigation, these trends suggest that structural disorder may contribute to improved stability by enabling a more adaptable catalyst structure under anodic conditions. Although the performance divergence remains limited within the investigated timeframe, such differences could become increasingly relevant over extended operating periods, emphasizing the importance of considering structural order, in addition to composition and activity, in the design of NiFe‐based OER electrocatalysts for AEMWE.

## Supporting Information

Additional supporting information can be found online in the supporting information section.

## Conflicts of Interest

The authors have no conflict of interest.

## Supporting information

Supplementary Material

## Data Availability

The data that support the findings of this study are available from the corresponding author upon reasonable request.
