# Supplementary material for: Impact of Crystallinity and Ni–Fe Composition on the Oxygen Evolution Performance of Ni x Fe1‐x O Electrocatalysts in Anion Exchange Membrane Water Electrolysis
Source: ChemSusChem. 2026 May 15;19(10):e202502755. doi: 10.1002/cssc.202502755 (PMC13177171; doi:10.1002/cssc.202502755)
Supplement: Supplementary file 1 — Supplementary Material [file CSSC-19-e202502755-s001.pdf]

**Supporting Information**

# Impact of Crystallinity and Ni–Fe Composition on the Oxygen Evolution Performance of $Ni_xFe_{1-x}O$ electrocatalysts in AEM Water Electrolysis

Atta Muhammad[1,2,3], Tatiana Rodriguez-Flores[1], Mohsin Muhyuddin[1,2], Fabio Di Fonzo[3], Enrico Berretti[4], Alessandro Lavacchi[4], Carmelo Lo Vecchio[5], Irene Gatto[5], Vincenzo Baglio[5], *Roberto Nisticò[1], **Carlo Santoro[1,2]

[1] Department of Materials Science, University of Milano-Bicocca, U5, Via R. Cozzi 55, 20125, Milano, Italy
[2] Electrocatalysis and Bioelectrocatalysis Laboratory, University of Milano-Bicocca, Via R. Cozzi 55, 20125, Milano, Italy
[3] X-nano s.r.l. Via Rubattino 81, 20134 Milan, Italy
[4] Istituto di Chimica Dei Composti OrganoMetallici (ICCOM), Consiglio Nazionale Delle Ricerche (CNR), Via Madonna Del Piano 10, Sesto Fiorentino, 50019, Firenze, Italy
[5] CNR-ITAE, Istituto di Tecnologie Avanzate per l'Energia "Nicola Giordano", Salita Santa Lucia sopra Contesse, 5, 98126, Messina, Italy

**Corresponding authors:**
* Roberto Nisticò: email: roberto.nistico@unimib.it
** Carlo Santoro: email: carlo.santoro@unimib.it

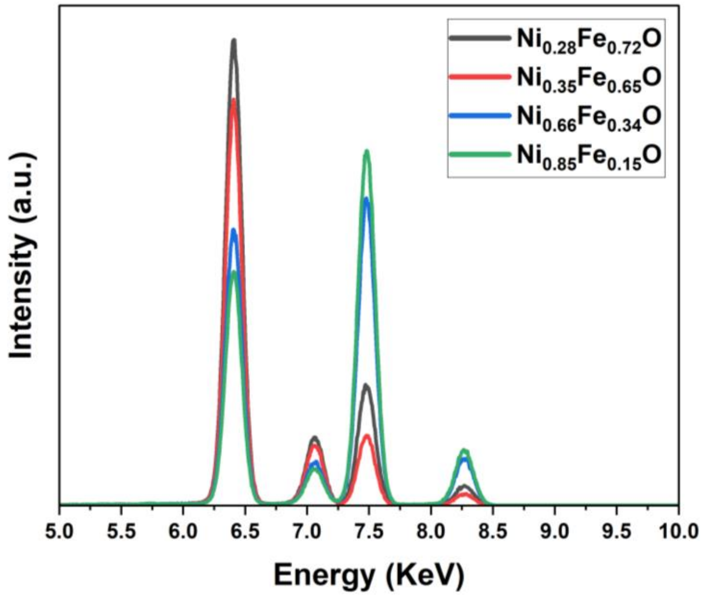


**Figure S1.** XRF spectra of the as developed $Ni_xFe_{1-x}O$ materials

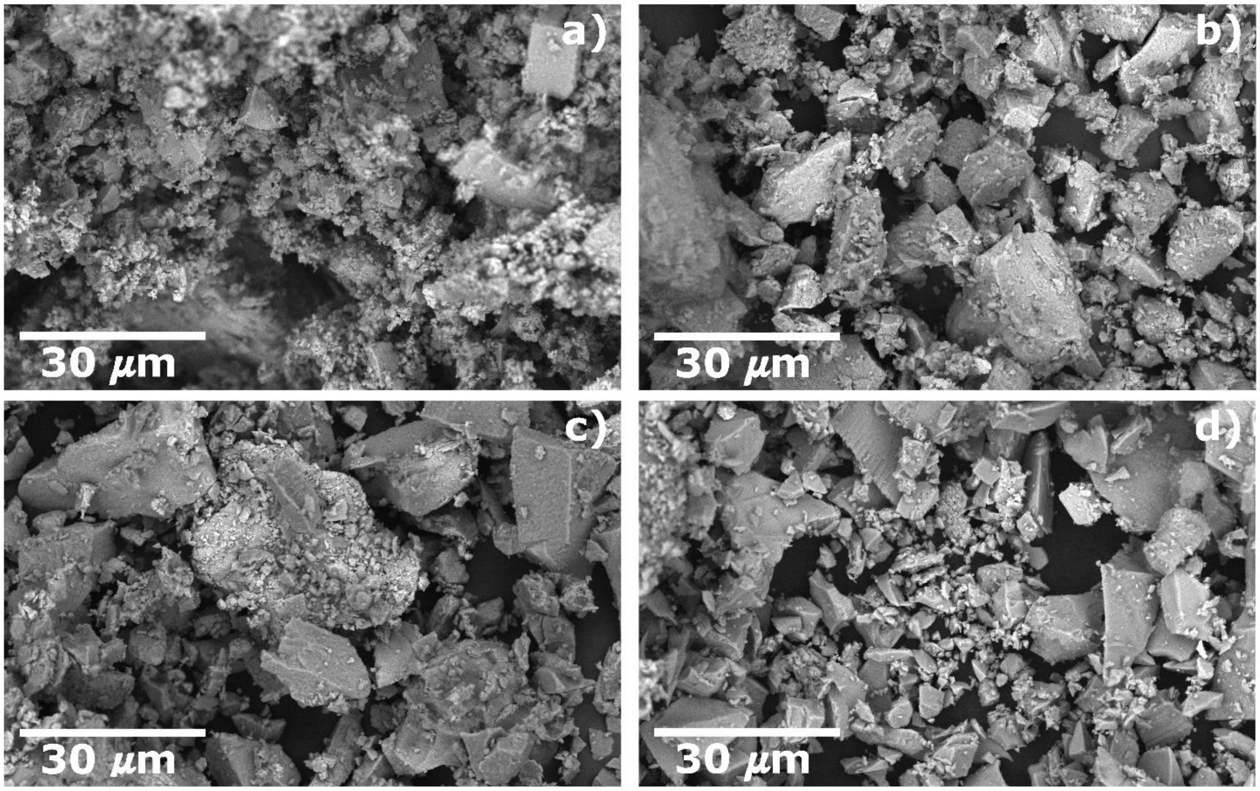


**Figure S2.** SEM Images of **(a)** $Ni_{0.28}Fe_{0.72}O$, **(b)** $Ni_{0.66}Fe_{0.34}O$, **(c)** $Ni_{0.85}Fe_{0.15}O$ and **(d)** $Ni_{0.35}Fe_{0.65}O$

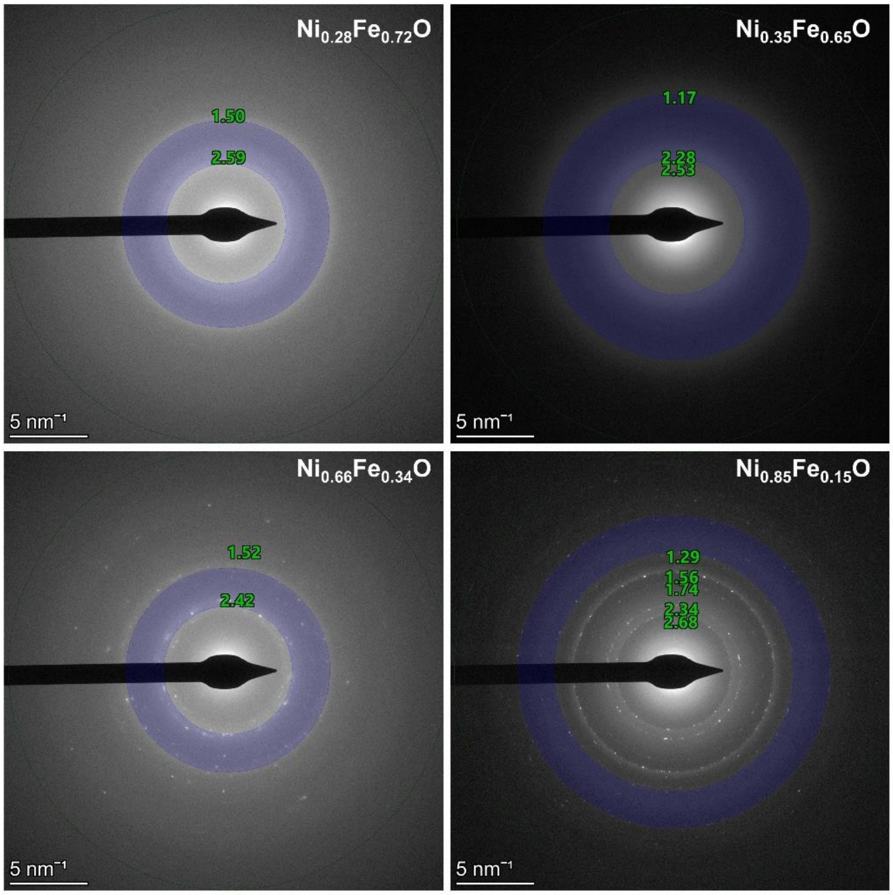


**Figure S3.** SAED Patterns of various $Ni_xFe_{1-x}O$ materials

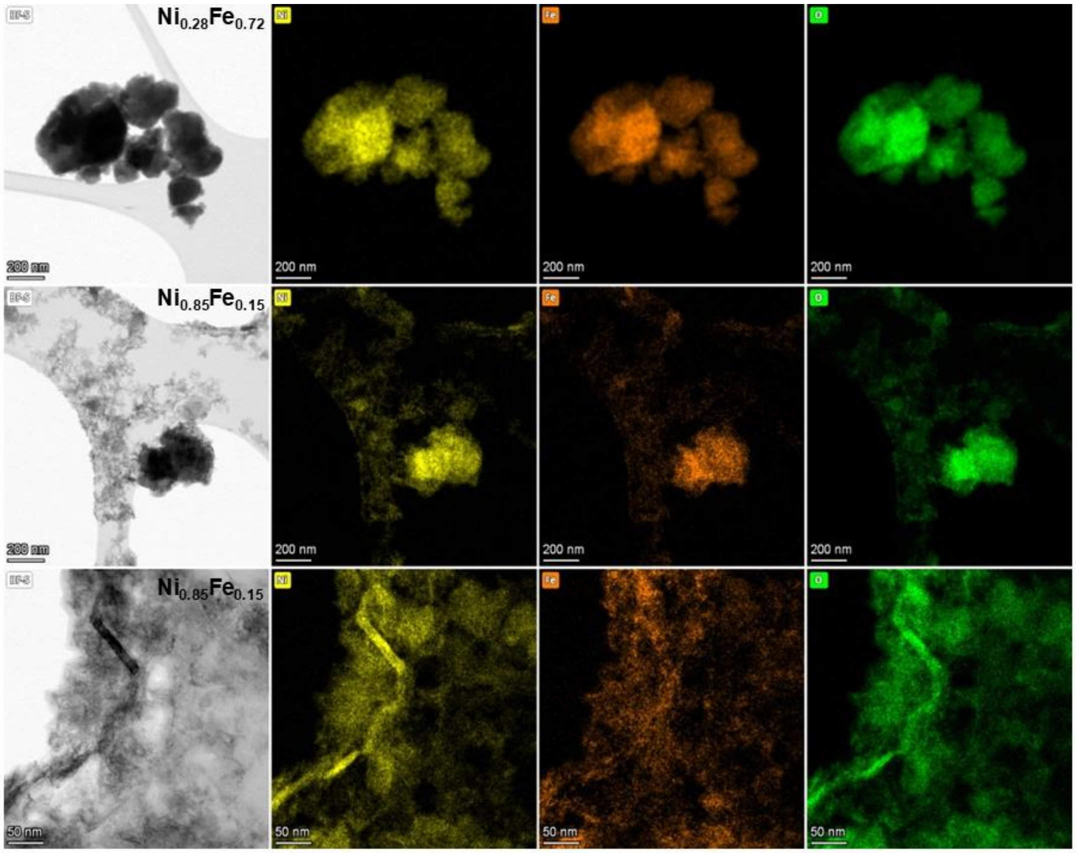


**Figure S4.** STEM images of samples 1st Row $Ni_{0.28}Fe_{0.72}O$, and 2nd and 3rd for $Ni_{0.85}Fe_{0.15}O$ from left to right columns: Bright field images, Ni map, Fe Map, O map

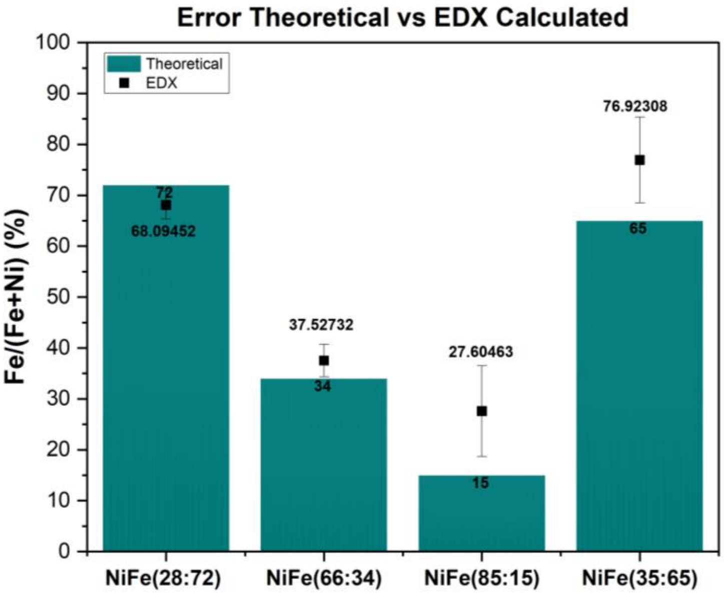


**Figure S5.** EDX Fe atomic ratio and error bars respect to theoretical values for all analysed

samples.

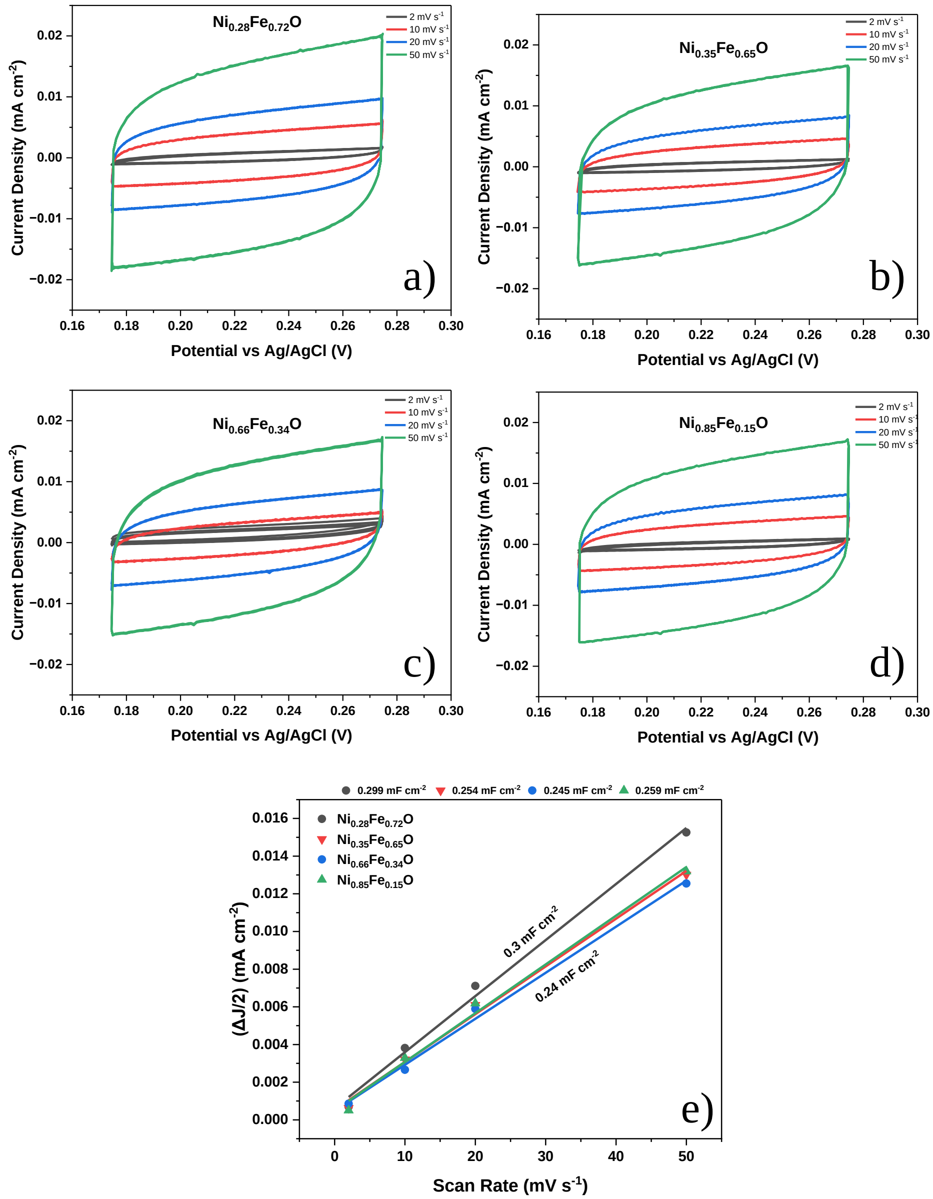


**Figure S6.** Cyclic voltammetry curves (non-faradic region) **a)** $Ni_{0.28}Fe_{0.72}O$ **b)** $Ni_{0.35}Fe_{0.65}O$ **c)** $Ni_{0.66}Fe_{0.34}O$ **d)** $Ni_{0.85}Fe_{0.15}O$ **e)** $C_{dl}$ plots for $Ni_XFe_{1-x}O$ catalysts

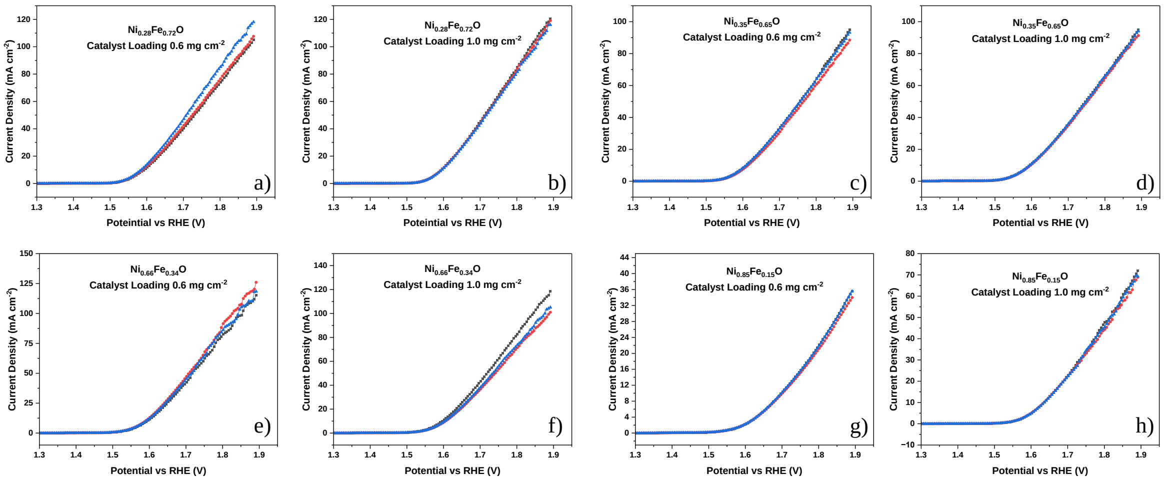


**Figure S7.** Polarization curves at different loadings **a)** $Ni_{0.28}Fe_{0.72}O$ at 0.6 mg cm$^{-2}$ **b)** $Ni_{0.28}Fe_{0.72}O$ at 1.0 mg cm$^{-2}$ **c)** $Ni_{0.35}Fe_{0.65}O$ at 0.6 mg cm$^{-2}$ **d)** $Ni_{0.35}Fe_{0.65}O$ at 1.0 mg cm$^{-2}$ **e)** $Ni_{0.66}Fe_{0.34}O$ at 0.6 mg cm$^{-2}$ **f)** $Ni_{0.66}Fe_{0.34}O$ at 1.0 mg cm$^{-2}$ **g)** $Ni_{0.85}Fe_{0.15}O$ at 0.6 mg cm$^{-2}$ **h)** $Ni_{0.85}Fe_{0.15}O$ at 1.0 mg cm$^{-2}$

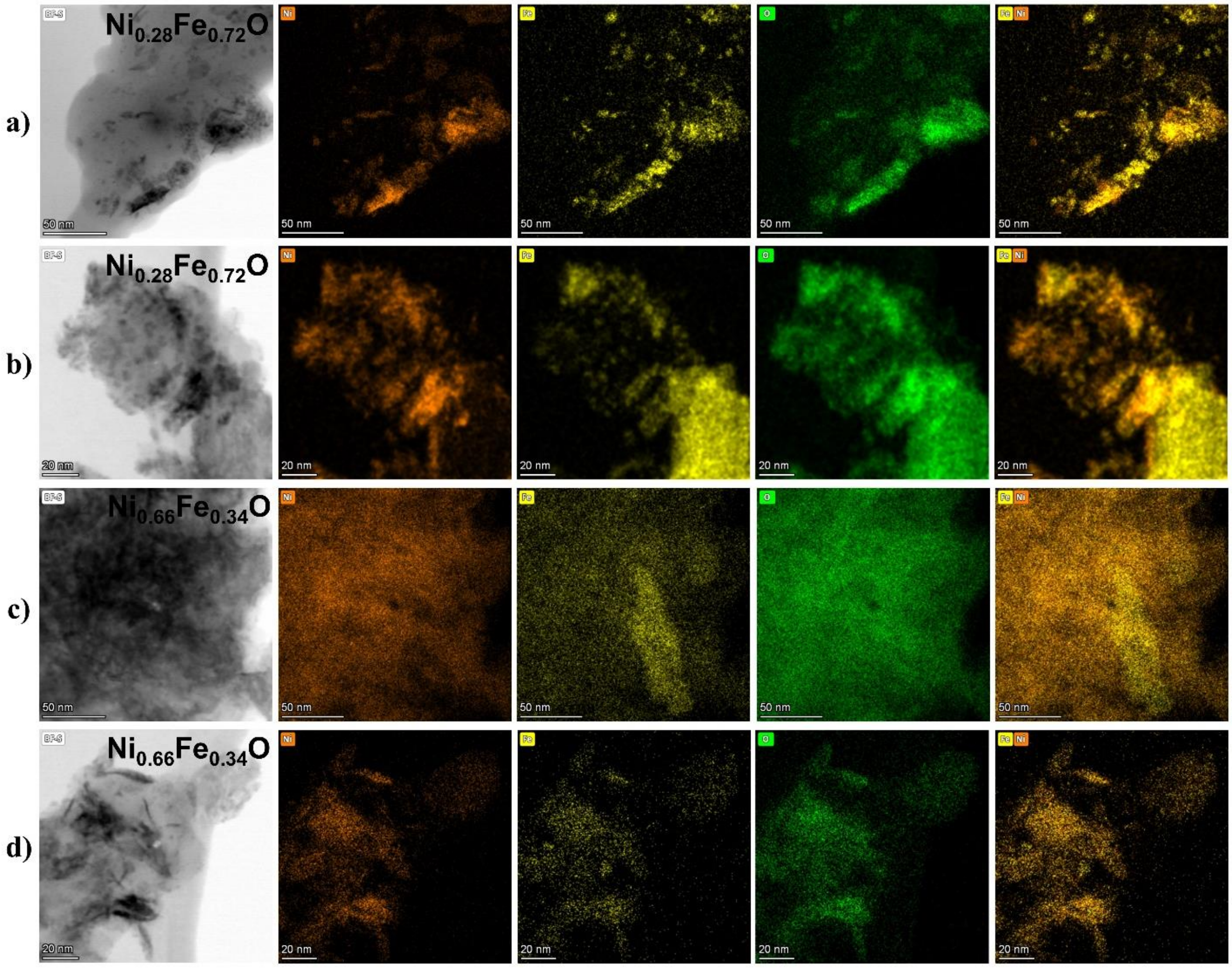


**Figure S8**. STEM images of post-OER samples at low and high magnification a) $Ni_{0.28}Fe_{0.72}O$ (50nm) , b) $Ni_{0.28}Fe_{0.72}O$ (20nm) c) $Ni_{0.66}Fe_{0.34}O$ (50nm), d) $Ni_{0.66}Fe_{0.34}O$ (50nm), from left to right columns: Bright field images, Ni map, Fe Map, O map and Ni-Fe map

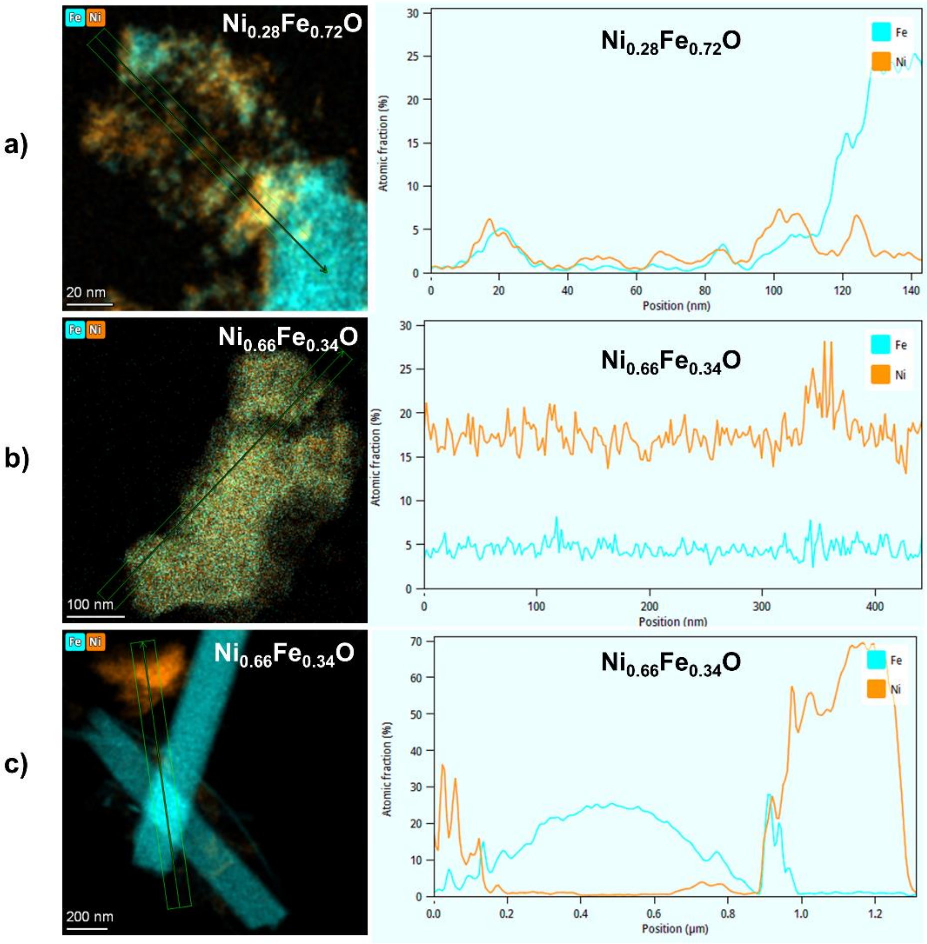


**Figure S9**. Post-OER STEM-EDS for $Ni_{0.28}Fe_{0.72}O$ vs $Ni_{0.66}Fe_{0.34}O$. a) HAADF-STEM $Ni_{0.28}Fe_{0.72}O$ (20 nm) and EDS line profile **b)** HAADF-STEM and EDS line profile for $Ni_{0.66}Fe_{0.34}O$ **c)** HAADF-STEM and EDS line profile for pillar-like $FeO_x$ domains in $Ni_{0.66}Fe_{0.34}O$ EDS-Line Profile the % is related to atomic % of the two Ni and Fe, considering also C and O to reach 100%

**Table S1.** Overpotentials for $Ni_XFe_{1-x}O$ catalysts at varying loadings and LSV replicates.

| Sample Name | Catalyst Loading mg $cm^{-2}$ | Overpotentials (η) (mV) at 10 mA $cm^{-2}$ (LSV-01) | Overpotentials (η) (mV) at 10 mA $cm^{-2}$ (LSV-02) | Overpotentials (η) (mV) at 10 mA $cm^{-2}$ (LSV-03) | Mean Value | Standard Deviation |
|---|---|---|---|---|---|---|
| $Ni_{0.28}Fe_{0.72}O$ | 0.6 | 362.3 | 359.8 | 354.5 | 359.02 | 3.71 |
| | 1.0 | 364.9 | 363.9 | 364 | 364.27 | 0.55 |
| $Ni_{0.35}Fe_{0.65}O$ | 0.6 | 378.2 | 384 | 378.7 | 380.29 | 3.21 |
| | 1.0 | 365.5 | 369.2 | 368.3 | 367.66 | 1.93 |
| $Ni_{0.35}Fe_{0.65}O$ | 0.6 | 359.5 | 359.2 | 358.7 | 359.13 | 0.40 |
| | 1.0 | 374.4 | 373.6 | 366.5 | 371.48 | 4.34 |
| $Ni_{0.85}Fe_{0.15}O$ | 0.6 | 468.4 | 468 | 468.1 | 468.17 | 0.21 |
| | 1.0 | 405.1 | 404 | 405.8 | 404.96 | 0.91 |

**Table S2.** Charge transfer resistance ($R_{ct}$) and Series resistance ($R_s$) and of the $Ni_{0.66}Fe_{0.34}O$ and $Ni_{0.28}Fe_{0.72}O$ based AEMWE cell at various temperatures.

| **Sample** | **Temperature (°C)** | **$R_s$ (Ω cm²)** | **$R_{ct}$ (Ω cm²)** |
|---|---|---|---|
| $Ni_{0.66}Fe_{0.34}O$ | 40 | 0.088665 | 0.344337 |
| | 60 | 0.064079 | 0.166773 |
| | 80 | 0.053714 | 0.083653 |
| $Ni_{0.28}Fe_{0.72}O$ | 40 | 0.088498 | 0.238559 |
| | 60 | 0.057989 | 0.12395 |
| | 80 | 0.047543 | 0.085689 |
